# Ubiquitous Computing for Remote Cardiac Patient Monitoring: A Survey

**DOI:** 10.1155/2008/459185

**Published:** 2008-06-30

**Authors:** Sunil Kumar, Kashyap Kambhatla, Fei Hu, Mark Lifson, Yang Xiao

**Affiliations:** ^1^Department of Electrical and Computer Engineering, San Diego State University, San Diego, CA 92182, USA; ^2^Computational Science Research Center, San Diego State University, San Diego, CA 92182, USA; ^3^Department of Electrical and Computer Engineering, The University of Alabama, Tuscaloosa, AL 35487, USA; ^4^Department of Computer Engineering, Rochester Institute of Technology, Rochester, NY 14623, USA; ^5^Department of Computer Science, The University of Alabama, Tuscaloosa, AL 35487, USA

## Abstract

New wireless technologies, such as wireless LAN and sensor networks, for telecardiology purposes give new possibilities for monitoring vital parameters with wearable biomedical sensors, and give patients the freedom to be mobile and still be under continuous monitoring and thereby better quality of patient care. This paper will detail the architecture and quality-of-service (QoS) characteristics in integrated wireless telecardiology platforms. It will also discuss the current promising hardware/software platforms for wireless cardiac monitoring. The design methodology and challenges are provided for realistic implementation.

## 1. INTRODUCTION

Ubiquitous cardiac monitoring can be defined as *mobile computing, medical sensor, and communications technologies for monitoring cardiac patients*. This represents the evolution of e-health systems from traditional desktop telemedicine platforms to wireless and mobile configurations. Ubiquitous cardiac monitoring is part of telemedicine research [1–134]. Current and emerging developments in wireless communications integrated with developments in pervasive and wearable biomedical sensor technologies would have a radical impact on future health-care delivery systems. They would provide a solution to the problems such as access to care for large segments in the population, continuing healthcare cost inflation, and uneven geographic distribution of quality. Distribution of quality is improved by enhancing accessibility to care for underserved populations, containing cost inflation, and improving quality through the continuous care of patients.

On the other hand, we have the following requirements for the patient monitoring applications: (i) periodic transmission of routine vital signs and transmission of alerting signals when vital signs cross a threshold, patients cross a certain boundary, or device battery drops below a level, (ii) addressing many usability issues including user comfort and trust by monitoring wireless and mobile networks including the use of wearable, portable, or mobile devices, (iii) diversity of patients including those suffering from mental illnesses is likely to make patient monitoring by wireless networks a challenge because of possible paranoia related to hand-held or wearable wireless devices, (iv) comprehensive and high-speed access to wireless networks, reliable and scalable wireless infrastructure, secure and fast databases, and utilization of network intelligence and information, (v) amount and the frequency of information that needs to be transmitted is another challenge. Especially, for the cardiac monitoring systems, we have the following few requirements: (a) maintaining periodic transmissions of routine heart-beating signs and real-time transmission of alert/alarm, (b) creating wearable portable or mobile devices that provide a user with comfort, (c) accommodating the diversity amongst patients, (iv) providing comprehensive and high-speed access to wireless networks, reliable and scalable wireless infrastructure, secure and fast databases, (d) utilizing network intelligence and information for calculations, (e) varying the amount and the frequency of information that needs to be transmitted is another challenge. For example, some patients require that certain vital signs be transmitted every few minutes, while for others continuous monitoring every few seconds is necessary. For all patients, any major changes in the vital signs should be transmitted immediately.

Continuous cardiac monitoring, through wireless technology, can be used to follow up with patients that have survived cardiac arrest, ventricular tachycardia, or cardiac syncope as well as for diagnostic purposes in patients with diffuse arrhythmia. As PDA devices are reasonably priced and are becoming increasingly powerful, telemedicine systems using PDAs are a cost-effective solution in a range of medical applications and suitable for home care usage. Some currently used patient operated ambulatory ECG recording equipments, such as holters and transtelephonic devices, are time consuming to setup and suffer from poor patient compliance. Patients undergoing acute myocardial infarction and subsequent arrhythmia ending in ventricular fibrillation and sudden cardiac arrest will not be able to operate such equipment. For reliable monitoring, it is necessary to develop a completely automatic recording and analyzing unit, which can detect cardiac abnormalities and automatically send data and alarm(s) to a health provider or central alarm system. It has been clearly documented that time is a critical factor in order to perform early cardiopulmonary resuscitation (CPR) and early defibrillation, and if the time to start CPR after a cardiac arrest increases, the chance of survival is reduced [4].

New wireless technology for telecardiology purposes give new possibilities for monitoring vital parameters with *wearable* biomedical sensors, and give the patient the freedom to be mobile and still be under continuous monitoring and thereby better quality of patient care. The implications and potential of *wearable* cardiac monitoring technologies are paramount, since they could (i) detect early signs of health deterioration, (ii) notify health care providers in critical situations, (iii) find correlations between lifestyle and health, (iv) bring sports conditioning into a new dimension by providing detailed information about physiological signals under various exercise conditions, and (v) provide doctors with multisourced, real-time physiological data [1]. They will therefore give the patient the freedom to be mobile and still be under continuous monitoring in different environments such as both inside and outside homes, offices, hospitals, nursing homes, and traveling. Furthermore, context-awareness can enable personalized functions such as reminder services or medication support depending on measured vital signs and individual disease patterns.

In order to make wearable devices practical, a series of technical, legal, and sociological obstacles should be overcome. The major design requirements of the mobile unit are the following: (1) portable and lightweight, (2) power autonomy of a few hours, (3) user-friendly interface, (4) collection and display of critical biosignals, (5) very low failure rate and highly accurate alarm triggers, especially if used for diagnostic purposes, and (6) reliable real-time wireless data transmission any-time anywhere in any form.

In this paper, we will first introduce general requirements of ubiquitous computing telecardiology platforms in Section 2. Section 3 will discuss existing systems. The architecture of heterogeneous wireless platforms is stated in Section 4. Quality-of-service (QoS) analysis in mobile telecardiology systems is given in Section 5. The fourth-generation (4G) system is discussed in Section 6. Finally, Section 7 provides the prospective of next-generation (xG) telecardiology systems.

## 2. UBIQUITOUS COMPUTING FOR TELECARDIOLOGY

Ubiquitous computing, also called pervasive computing, is a concept for future computing environments where a user is surrounded by a large amount of small application-specific, network-connected information appliances. It is characterized by (i) physical and cognitive embedded systems, (ii) networking, (iii) ubiquity, (iv) context awareness, and (v) application specific devices (appliances). The costs for integrating the necessary communication abilities are affordable compared to the potential for cost savings and to the medical advantages: (i) context-aware, cognitive-embedded systems result in a better usability, which is essential for the use in a home-monitoring scenario (the device is adapting itself to the user and not vice versa), (ii) patient mobility is maximized by physically embedding the devices into wearable or implantable distributed systems, and (iii) networking compensates decentralization. Thus high performance services, such as pattern recognition with neural networks, can be provided anywhere to the mobile devices.

Problems emerge in ubiquitous computing in the following areas.

1. *Embedded* [39]:
  - both vital sensors and base station have to be wearable to ensure mobility and attention;
  - attention has to be paid to the body placement, human movement, weight, size, and other constraints;
  - reduce the power consumption relatively with the increase in the performance of processors, memories, and other components.
2. *Networking* [40, 41]:
  - providing ubiquitous access to central information services;
  - lower power consumption;
  - ensuring high level of security;
  - extensive interoperability features;
  - integrated functionality such as service discovery, self-configuration, encryption, and authentication;
  - protection of privacy and communication security considering mobile Internet connections.
3. *Context-awareness* [42]:
  - personal identifications, personalized health monitoring systems provide functions such as reminder services or medication support depending on measured vital signs and individual disease patterns. The data therefore should travel reliably to the remote nurse/physician;
  - time, a very simple but important context used for the timing synchronization of distributed sensors.

Wireless and mobile technologies are finding a role in cardiac patient monitoring in several different environments: homes, hospitals, and nursing homes. However, due to several limitations including unpredictable and spotty coverage of users by wireless networks, the quality and reliability of patient monitoring have not been highly satisfactory.

Wireless cardiac patient monitoring also raises several issues: (i) introduction of wireless technologies in patient monitoring is very preliminary as patient monitoring requirements and challenges in different environments have not been identified, (ii) unique capabilities of wireless and mobile infrastructure for patient monitoring have not been utilized, (iii) applications and solutions for patient monitoring are limited to using a single type of wireless network, thus restricting the access and coverage, and (iv) introduction of wireless and mobile technologies in patient monitoring is very fragmented and limited to few simple cases.

For some patients, certain vital signs need to be transmitted every few minutes, while for others continuous monitoring every few seconds is necessary. For all patients, any major changes in the vital signs should be transmitted immediately. In wireless environment, it could be better to transmit differential changes since the last time or a reference value to reduce the amount of information. This would also increase the chances of reception by others. An increasing number of patients will be wearing portable or mobile devices that would have access to public or private wireless networks. To improve the quality and reliability of patient monitoring, these devices could be designed to operate in ad hoc wireless mode. Also with a lack of coverage and variable fluctuating coverage by infrastructure-oriented wireless networks in some spots combined with patients in locations not reachable by others, an increased reliability of monitoring and higher chances of transmission of alert signal can be achieved with the use of ad hoc wireless networks. Due to power and size requirements of these devices, the range of transmitted signal is likely to be small. Further, the range is likely to be affected both by the frequency of operation and the nature of spectrum used (licensed versus unlicensed). For monitoring of patients who are not covered by an infrastructure-oriented wireless networks, the cooperation of others with wireless devices in an ad hoc configuration will be utilized.

Increasing life expectancy accompanied with decreasing dependency ratio in developed countries calls for new solutions to support independent living of the elderly. *Ubiquitous computing* technologies can be used to provide these solutions so that they naturally support the elderly and their caregivers in their everyday environment. While designing these solutions, special attention should be paid on users' needs and abilities and on the way the solutions fit to the provisioning of the care. Finally, any solution should be properly validated and tested in real environments with a sufficient number of real users.

Another concept, called *mobile health (m-health),* has a similar definition as the above system. Current and emerging developments in wireless communications integrated with developments in pervasive and wearable technologies would have a radical impact on future health-care delivery systems. A snapshot of recent developments in these areas and some of the challenges and future implementation issues from the m-health perspective are presented and addressed in [43]. A comprehensive overview of some of these existing wireless telemedicine applications and research can be found in recent publications in the area [44–47, 118–134].

There are some limitations to existing wireless technologies that mostly depend on general packet radio service (GPRS) technologies and on their deployment strategies in health care. Some of these issues can be summarized as follows [48]. (1) The lack of an existing flexible and integrated “m-health-on-demand” linkage of the different mobile telecommunication options and standards for e-health services. This lack of linkage and compatibility for telemedical services exists due to the difficulty of achieving operational compatibility between the telecommunication services, terminals, and devices standards, and “m-health protocols.” (2) The high cost of communication links, especially between satellites and global mobile devices, and the limitation of existing wireless data rates especially for the globally available 2.5G and third-generation (3G) services for some e-health services. This is also combined with the availability of secure mobile Internet connectivity and information access especially for e-health systems. (3) Health-care is a very complex industry that is difficult to change. Organizational changes are very often required for health-care institutions to benefit from e-health and m-health services. (4) The short-term and long-term economic consequences and working conditions for physicians and health-care experts using these technologies are not yet fully understood or properly investigated. (5) The methods of payment and reimbursement issues for e-health and m-health services are not yet fully developed and standardized. (6) There is a lack of integration between existing e-health services and other information systems (e.g., referral and ordering systems, medical records, etc.). (7) The demonstration projects so far have failed to show that m-health services result in real savings and have cost effective potential.

Mobile wireless technologies such as mobile computing, wireless networks, and global positioning systems (GPS) have been employed in several countries for emergency as well as general health care [49, 50]. Though the systems show promising research in using WLAN radios to transfer patient information to the hospital in real time, analyzing signal interference with other available Wi-Fi hotspots, security of medical data, transmission in areas devoid of Wi-Fi hotspots has not been addressed significantly.

Complete reliance on cellular architecture could prove futile in some locations where the cellular coverage is either deteriorated significantly due to many factors or is totally unavailable as in rural areas. In [51], excessive complex computations and frequently large data transmissions could put serious restrictions on the energy utilization of mobile phone. Also significant interference alleviation of the Bluetooth module with other networks, such as Wi-Fi and personal area networks, has not been addressed.

Current 3G cellular and WLAN networks experience signal attenuation/loss due to fading, multiradio interference and multipath distortion. Fading can be because of interference from other radio signals present in the same part of the spectrum as well as because of moving equipment. Dead spots may occur within buildings due to signal attenuation by the construction material and metals, creating a dead spot in its radio shadow. Typical examples of dead spots include subway train platforms, indoor environments, and underground areas. The spontaneous loss of communications for no apparent reason is probably one of the most irritating aspects of various wireless networks.

Moreover, subscribers experience higher call blocking in dense areas known as hot spots, such as downtown areas and amusement parks. A costly solution is to install more base stations or access points in such regions. Alternatively, multihop relaying in ad hoc networks can be used to provide communication in dead spot and hot spot regions, without having to depend on an existing central controller or an infrastructure. Users in dead spot areas can maintain connectivity with the base station (BS) by relaying their messages through other subscribers or by hopping to a user who has a connection with the BS. In hot spots a user can obtain a connection by hopping away from congested BSs to lightly loaded ones. Relaying in cellular networks solves the coverage and high data rate problems, reduces peak power consumption, and provides load balancing in dense and highly populated areas. In fact, due to these potential benefits, there has recently been an interest in deploying this technique in 3G cellular systems as discussed below. Efforts in developing heterogeneous networks by interconnecting these technologies have opened up new areas of research.

## 3. OVERVIEW OF EXISTING MOBILE TELECARDIOLOGY SYSTEMS

In this section, we provide an overview of technologies used for transmitting the vital physiological data from a cardiac patient to a remote monitoring station.

The European EPI-MEDICS project [24] uses an intelligent personal ECG monitor (PEM) for the early detection of cardiac ischemia and arrhythmia. The PEM records a 3-lead ECG (DI, DII, and V2), derives the corresponding standard 12-lead ECG using a patient specific transform [25], and stores the information in patient's electronic health record (EHR) on a smart media card in the PEM device. The PEM generates different levels of alarms based on the ECG and the patient’s clinical history, and forwards them with the recorded signals and the EHR to the health care providers by means of Bluetooth and GSM/GPRS in store and forward mode [26].

The fast development of mobile technologies, including increased communication bandwidth and miniaturization of mobile terminals, has accelerated developments in the field of mobile telecardiology [2]. Development of the efficient portable and body-worn devices and systems to measure physiological parameters of cardiac patients (e.g., ECG, heart rate, SpO2, and blood pressure) has been witnessing active research lately. A wireless PDA-based physiological monitoring system for patients inside the hospital was reported in [3] which transmits the acquired data to a central management unit by using the hospital WLAN.

A system for out-of-hospital follow-up and monitoring of patients with chronic heart disease was discussed in [29]. The patients belonged to one of four risk groups: arterial hypertension, malignant arrhythmias, heart failure, and post-infarction rehabilitation. They were provided with portable recording equipment and a cellular phone that supported ECG data transmission and wireless application protocol (WAP). In order to avoid hospitalization for purely health monitoring purpose, researchers at the University of Karlsruhe are developing a remote personal health monitoring system by employing a platform consisting of wearable smart sensors measuring vital signs and of a base station communicating wirelessly with the sensors [30, 31]. A patient monitoring system using multihop ad hoc wireless networks is presented in [32], in which vital signs from one patient are forwarded to another patient and this could then be transferred to an access point within range.

The patients with an artificial heart implant are discharged from the hospital after an appropriate stabilization period. A web-based database system for reliable intelligent continuous remote monitoring of such patients was developed in [34]. The system consists of a portable/desktop monitoring terminal, a database for continuous recording of patient and device status, and an “intelligent diagnosis algorithm module” that noninvasively estimates blood pump output.

### 3.1. Using wearable sensors

Among commercial telemetry systems, CardioNet is the first provider of mobile cardiac outpatient telemetry (MCOT) service in USA for continuous monitoring of patient’s ECG and heartbeat, at home, at work, or traveling [14, 15]. It automatically detects and transmits (using cellular connection) abnormal heart rhythms to the CardioNet monitoring center, where certified cardiac technicians analyze the transmissions and respond appropriately 24/7/365. In a clinical study, CardioNet mobile cardiac outpatient telemetry detected serious arrhythmias in 53% of patients who had been previously monitored with Holter and/or event recording where no arrhythmia was detected. Philips provides a telemetry system, which consists of a portable TeleMon companion monitor and the IntelliVue Information Center to offer an integrated surveillance and monitoring solution for ambulating patients who require vigilant oversight of ECG and SpO2 [16]. The IntelliVue telemetry system uses a cellular network to provide reliable two-way communications between transceivers and the Information Center. The system provides audible feedback from transceiver on SpO2 spot checks and patient out of range. The GMP Wireless Medicine Corp. developed LifeSync wireless ECG system for bedside monitoring [17, 18].

A wearable wireless biomedical sensor system has been developed in [5]. The patient wears a wireless ECG sensor, which transmits ECG signals to a dedicated hand-held device (HDD). If an abnormal cardiac signal is encountered, the HHD unit transmits (using 3G cellular phone) the recorded ECG to a hospital with an alarm. The system uses an error correction protocol based on the TCP/IP for reliable transmission. However, the system prototype seems to be too large for routine use by outpatients.

An innovative system named WEALTHY is presented in [6], where conducting and piezoresistive materials in form of fiber and yarn are integrated in a garment and used as sensor and electrode elements for simultaneous recording of vital signs. Microsoft Corporation has developed “HealthGear,” a real-time wearable system consisting of a set of physiological sensors wirelessly connected via Bluetooth to a Bluetooth-enabled cell phone for monitoring and analyzing physiological signals [1]. Some other wearable health monitoring devices have been discussed in [7–12]. The MobiHealth project in Europe aims to provide continuous monitoring of patients outside the hospital environment by using a 3G-enabled body area networks (BAN) [13]. The major issues considered in the MobiHealth project are security, availability, and reliability of communication resources and QoS guarantees for bandwidth, delay, and bit error rate.

A wireless and wearable ECG monitoring system was proposed in [27] to detect the rare occurrences of cardiac arrhythmias and to follow up critical patients from their home. It continuously measures and transmits the sampled ECG signals to the patient’s PDA using a built-in RF-radio transmitter. The PDA automatically connects to a GPRS mobile network to transmit the data to health provider. A real-time patient monitoring system that integrates vital signs sensors, location sensors, ad hoc networking, electronic patient records, and web portal technology was designed and developed in [28].

The design of a processor, which samples signals from sensors on the patient, has been addressed in [37]. The processor consists of a signal conditioning module, a peripheral control module, a processor and memory module with a microcontroller, and a Bluetooth communication module. In Finland, a system with secure mobile healthcare services was tested in 2003 [38].

CodeBlue is a wireless infrastructure for emergency medical care integrating low-power wireless vital sign sensors, PDAs and PCs [19, 20]. Some of their research interests include the integration of medical sensors with low-power wireless networks, wireless ad hoc routing protocols, and adaptive resource management. The system is scalable and robust with respect to the number of simultaneous queries, data rates, and transmitting sensors. Some other critical issues considered by CodeBlue are flexible device discovery and naming for sensors, prioritization of patient data, security, and fast and reliable tracking of rescuer and victim locations. The AMON system is a wrist-worn medical monitoring and alert system targeting high-risk cardiac/respiratory patients [21]. The system includes continuous collection and evaluation of multiple vital signs, medical emergency detection, and GSM/UMTS cellular connection to a medical center.

**More recent advances in wearable medical sensors**

A new system-on-a-chip radar sensor for next-generation wearable wireless interface applied to the human health-care and safeguard is presented in [135]. The overall system consists of a radar sensor for detecting the heart and breath rates and a low-power IEEE 802.15.4 ZigBee radio interface, which provides a wireless data link with remote data acquisition and control units. Particularly, the pulse radar exploits 3.1–10.6 GHz ultrawide band signals, which allow a significant reduction of the transceiver complexity and, then, of its power consumption. Such a novel system-on-a-chip wireless wearable interface enables low-cost silicon technologies for contactless measuring of the primary vital signs and extends the capability in terms of applications for the emerging wireless body area networks.

Reference [136] presents a compact planar antenna designed for wireless sensors intended for healthcare applications. Antenna performance is investigated with regards to various parameters governing the overall sensor operation. The study illustrates the importance of including full sensor details in determining and analyzing the antenna performance. A globally optimized sensor antenna shows an increase in antenna gain by 2.8 dB and 29% higher radiation efficiency in comparison to a conventional printed strip antenna. The wearable sensor performance is demonstrated, and effects on antenna radiated power, efficiency, and front-to-back ratio of radiated energy are investigated both numerically and experimentally.

Although it is suggested to use wearable medical devices and sensors as the nodes of body sensor networks (BSN) could allow better long-term monitoring of health condition, the protocol and criteria for validating these nodes in clinical settings are often overlooked. By using the validation of blood pressure (BP) measurement devices as an example, [137] reveals the need for new standards for nodes of BSN and proposes essential considerations to it. It is observed from a previous clinical survey and their theoretical analysis that there are disagreements in existing standards on the validation of conventional BP devices. Moreover, an experiment carried out by their group on 85 subjects demonstrates the inappropriateness of using the existing protocols that are setup for validating cuff-based BP devices to evaluate new cuff-less measurement techniques. The results suggested that a third objective measure could be introduced to relate the variant standards. It is also proposed that different validation criteria should be used for nodes of BSN that are developed based on new measurement principles.

Reference [138] analyzes the main challenges associated with noninvasive, continuous, wearable, and long-term breathing monitoring. The characteristics of an acoustic breathing signal from a miniature sensor are studied in the presence of sources of noise and interference artifacts that affect the signal. Based on these results, an algorithm has been devised to detect breathing. It is possible to implement the algorithm on a single integrated circuit, making it suitable for a miniature sensor device. The algorithm is tested in the presence of noise sources on five subjects and shows an average success rate of 91.3% (combined true positives and true negatives).

### 3.2. Using cell phones

Some researchers have used cellular phones to transmit the vital signs from the ambulance to the hospital, either in store-and-forward mode [22] or in real-time mode [23].

A 3G universal mobile telecommunications system (UMTS) solution for the delivery of voice, real-time video, ECG signals, and medical scans information from an ambulance to a hospital is presented in [33]. The quality-of-service constraints for different services are investigated and feasibility of using UMTS technology is demonstrated. However, the QoS provisioning is provided within the patient’s data only (ECG/video/voice) and not with other services in UMTS. The number of lost packets is very high as the number of users approaches 30. The system has to be evaluated for reliability measurement at high transmission rate requirements.

Steady advances in wireless networking, medical sensors, and interoperability software create exciting possibilities for improving the way we provide emergency care. The Advanced Health and Disaster Aid Network (AID-N) [35], being developed at Applied Physics Lab of Johns Hopkins University, explores and show cases of how these advances in technology can be employed to assist victims and responders in times of emergency. The system in [28] covers a subset of the technologies in AID-N.

A cost-effective portable teletrauma system that assists health-care centers in providing prehospital trauma care is developed and implemented in [36]. The simultaneous transmission of a patient’s video, medical images, and electrocardiogram signals, which is required throughout the prehospital procedure, is demonstrated over commercially available 3G wireless cellular data service. Physicians can remotely control the information sent from the patient side. This allows a trauma specialist to be virtually present at the remote location and participate in prehospital care, which can potentially reduce mortality and morbidity. To alleviate the limited and fluctuant bandwidth barriers of the wireless cellular link, the system adapts to network conditions through media transformations, data prioritization, and application-level congestion control methods.

A multihop relaying scheme is used as overlay architecture for single-hop time division duplex (TDD) wideband-code division multiple access (W-CDMA) cellular networks in [52]. A major challenge pertaining to the introduction of this technology into cellular networks is the design of an efficient slot assignment algorithm for relaying nodes. An heuristic slot assignment scheme, namely delay-sensitive slot assignment (DSSA), is proposed in [53]. DSSA is capable of fully utilizing a limited number of channels to enhance spatial reuse and reduce the end-to-end delay.

A third-generation universal mobile telecommunications system (UMTS) solution for the delivery of biomedical information from an ambulance to a hospital is presented in [115]. The joint transmission of voice, real-time video, electrocardiogram signals, and medical scans in a realistic cellular multiuser simulation environment is considered, taking into account the advantages and particularities of UMTS technology for such transmission. The accomplishment of quality-of-service constraints for different services is investigated and quantitative results were provided to demonstrate the feasibility of using UMTS technology for emergency care services on high-speed moving ambulance vehicles.

The evaluation of the above technologies points to a number of critical areas for future work. The most serious is the lack of reliable communication, although results show that this problem can be mitigated somewhat through redundant transmissions. Reliable routing may not be required for all medical data; rather, the system should allow each query to specify its reliability needs in terms of acceptable loss, data rate, or jitter. Another area worth exploring is the impact of bandwidth limitations and effective techniques for sharing bandwidth across patient sensors. For example, each CodeBlue query could specify a data priority that would allow certain messages (e.g., an alert from a critical patient) to have higher priority than others in the presence of radio congestion. This approach can be combined with rate-limiting congestion control [14, 25] to bound the bandwidth usage of patient sensors. An important shortcoming of the current CodeBlue prototype is its lack of security. Integration of private-key encryption [29] along with a public-key protocol for key distribution [22, 38] should be investigated. The privacy and security requirements for medical care are complex and differ depending on the scenario. For example, HIPAA privacy regulations need not be enforced during life-saving procedures. Nevertheless, integration of some forms of end-to-end security would be very helpful.

## 4. USING HETEROGENEOUS (CELLULAR, WLAN, AND AD HOC) NETWORKS

The advent of a myriad of wireless networking technologies allows a mobile host today to be equipped with multiple wireless interfaces that have access to different wireless networks. The present cellular network-based hybrid schemes to handle host mobility are heavily dominated by infrastructure-based schemes. The vertical handoffs [65, 66] assuming the support of Mobile IP [67] have been proposed to ensure continuous connectivity when the mobile host roams between different wireless networks. Various interworking architectures [68–70, 139, 140] have also been proposed to facilitate link-layer handoffs between the WLAN and cellular networks. Other approaches include providing a basic access network [71] or a communication gateway [72] that the mobile host can access directly without being exposed to the heterogeneity of the backend network infrastructure. In [73], an end-to-end approach (without relying on the infrastructure support) is proposed to handle connection migration when the network address of the mobile host changes during the lifetime of the connection.

The major drawbacks in handling host mobility in 3G cellular-based heterogeneous networks are as follows.

- *Reliance on infrastructure support*. Although several approaches have been proposed recently for achieving host mobility without incurring the overheads in Mobile IP [74–77], given the increasing heterogeneity of wireless access technologies, the effectiveness of these approaches is greatly limited by the specificity to the networks they are designed for.
- *Inability to leverage soft handoffs*. Even if soft handoffs are possible at the link layer, in the absence of any explicit support at a higher layer, the migrated connection experiences packet losses and suffers from performance degradation during the handoffs across heterogeneous networks. Specifically, although TCP migration [78] achieves host mobility without relying on the network support, it performs a “hard handoff” at the transport layer between the new and old TCP states (i.e., transmission control blocks [79]).
- *Lack of support for network-specific congestion control schemes*. Despite the availability of network-specific congestion control schemes, no existing solutions allow the mobile host to dynamically change them for a live connection when it migrates to a heterogeneous network.
- *No provision for resource aggregation*. When a multihomed mobile host is within reach of more than one network, existing approaches do not allow it to leverage their resources simultaneously.

An end-to-end scheme that enables a multihomed mobile host to seamlessly use the heterogeneous wireless access technologies is recently proposed in [80], which allows host mobility, seamless vertical handoffs, and effective bandwidth aggregation when the mobile host has simultaneous access to multiple networks.

In 3G cellular networks, mobile users experiencing poor channel quality usually have low data-rate connections with the base station. A unified cellular and ad hoc network (UCAN) architecture for enhancing cell throughput, while maintaining fairness, is proposed in [54]. In UCAN, a mobile client has both 3G cellular link and IEEE 802.11-based peer-to-peer link. The UCAN architecture improves individual user's throughput by up to 310% and the aggregate throughput of the HDR downlink by up to 60%. Several companies such as GTRAN Wireless [55] are offering integrated cards that implement both IEEE 802.11b and 3G wireless interfaces. Thus, if routing protocols can be made aware of both interfaces, they can improve performance significantly by selecting the best interface(s) to deliver packets to the mobile users.

Deficiencies of mobile ad hoc networks include limited wireless bandwidth efficiency, low throughput, large delays, and weak security. Integrating it with a cellular network can improve communication and security in ad hoc networks, as well as enrich the cellular services. A cellular-aided mobile ad hoc network (CAMA) architecture is proposed in [56], which provides throughput enhancements. A CAMA agent in the cellular network manages the control information, and the routing and security information is exchanged between mobile terminals (MTs) and the agent through cellular radio channels. A position-based routing protocol is developed in CAMA to make more accurate routing decisions. CAMA involves high GPS cost and incurs delay when talking to the CAMA agent. The integrated cellular and ad hoc relaying (iCAR) architecture addresses the congestion problem due to unbalanced traffic in a cellular system and provides interoperability for heterogeneous networks [57]. A limited number of ad hoc relaying stations (ARS) and some increase in the signaling overhead (as well as hardware complexity) reduce the call blocking/dropping probability in a congested cell and the overall system. A thorough performance study of two such novel throughput enhancement architectures, iCAR and multihop cellular network (MCN), is carried out in [58]. iCAR balances the traffic load and increases the network throughput by relaying excess traffic from a hot cell to cooler cells through a multihop path over special routers called ad hoc relaying stations (ARS). However, it requires large number of relay stations due to small coverage and cellular channel shared between relay station and base station. MCN increases the network throughput by reducing the data transmission power to half of the cell radius, thus increasing the spatial reuse of bandwidth. These two architectures thus benefit by combining the best of cellular and ad hoc systems.

Directional antennas were used to enhance the spatial reuse in the ad hoc-cellular (A-Cell) architecture [59]. In [60], the throughput performance of ad hoc GSM (A-GSM) scheme with respect to the number of dead spot locations, average dead spot size, and mobile node population has been investigated and compared to that of a 2G GSM system. Area coverage and capacity enhancement have also been investigated in [61], where the multihop relaying increases the area coverage by 40% compared to the single-hop case.

Correlated usage of mobile devices intensifies traffic bursts and introduces congestion in cellular networks. “PARCelS,” which utilizes roaming mobile hosts to perform route relaying, is discussed in [62]. “PARCelS” performs operations such as balancing traffic load, avoiding traffic congestion, and reducing latency. The disadvantages of PARCelS are the need for high mobile station density, complexity, and energy consumption.

Another hybrid network model of cellular and ad hoc networks called Sphinx is proposed in [63], which achieves higher throughput and lower power consumption. At the same time, Sphinx avoids the typical pitfalls of ad hoc networks including unfair resource allocation and throughput degradation due to mobility and traffic locality.

Users in a hot cell typically experience a large delay. In mobile assisted data forwarding (MADF) scheme [64], an ad hoc overlay network is added to the fixed cellular infrastructure and special channels called “forwarding channels” are used to connect users in a hot cell and its surrounding cold cells without going through the base station. The forwarding channel management in MADF is done by mobile units themselves. A major advantage in MADF is the low delay, but the need for reserved cellular channels at the relay station (i.e., mobile unit) increases the computational complexity.

## 5. QOS REQUIREMENTS IN MOBILE TELECARDIOLOGY APPLICATIONS

A comprehensive overview of various mobile telemedicine technologies as used in different settings is given in [81]. Based on its delay tolerance, the telemedicine data can be classified as stream (e.g., ambient video and audio), conversational (videoconferencing), near real-time, and not real-time. In medical environment, different data traffics with different classes of QoS requirements have to be transmitted simultaneously. One of the critical issues to be investigated is how to optimize the network performance to provide medical services with other multimedia services simultaneously.

The telehealth data from different patients, who may be widely scattered, will consist of various physiological parameters, text, voice as well as video for diverse chronic diseases. These data must coexist together as well as with other commercial data such as voice, video (streaming or real), multimedia, and Internet. QoS provisioning for the future advanced telehealth monitoring technologies and systems is therefore a difficult proposition as the communication infrastructure must satisfy QoS requirements of different classes of applications. In this section, we analyze the research carried out in QoS provisioning in past telehealth monitoring projects.

The wide development of multimedia clinical applications and the use of inter- and intrahospital communication networks require a specific analysis to increase healthcare services efficiency. A processing toolbox (QoSM3) for technical evaluation of quality-of-service (QoS) traffic requirements in new healthcare services based on telemedicine is proposed in [116]. This tool consists of the multimedia service definition and the measurement and modeling processes, which permit to analyze QoS requirements and to optimize application design regarding available network resources. The proposed methodology was tested to evaluate real-time and store-forward medical services.

With the advent of the “next-generation Internet” and various related technologies, important tradeoffs between emerging network capabilities and design-related application requirements for network-based distributed healthcare systems are observed in [117]. One of the aspects of these emerging network capabilities is quality of service (QoS). QoS guarantees are a necessary characteristic of “next-generation” networks which will have a profound impact on the deployment of advanced, network-sensitive medical applications. Some of the underlying aspects of QoS technologies and possible considerations in designing network-based medical systems are discussed in [117]. One such system that attempts to solve some QoS problems, as well as provides a system for telehealth, is called “Cardimon,” and will be overviewed in Section 7.

QoS guarantees are a necessary characteristic of “next-generation” networks, which will have a profound impact on the deployment of advanced, network-sensitive medical applications [82]. A mobile telemedicine system was tested in three ambulances using commercial, off-the-shelf equipment, with data-management and physician interface software [83]. The main problems encountered by the above prototype were the instability in the commercial system components and unpredictable wireless connectivity and bandwidth resulting in inconsistent system performance, which frustrates the physician. The project in [84] tested a patient monitoring system and evaluated the network performance. Although, the 3G networks significantly perform better than 2.5G, they might not be adequate to support the bandwidth and delay requirements of the sophisticated and demanding m-health services. The teletrauma scenario requires the transmission of patient’s vital signs together with audio/video signals in real-time from the scene of the accident [85]. High Plains Rural Health Network (HPRHN), based in Fort Morgan, Colorado, is an interactive video network for real-time monitoring and diagnosis of patients at the sites of serious automobile accidents on the highways. In [86], the notion of QoS for communication of telemedicine multimedia data is presented for preorchestrated as well as live videoconferences.

The InfraVIDA telemedicine system allows remotely located health professionals to make telediagnosis and get second medical opinion from consultants [87]. Applications get differentiated services (i.e., DiffServ QoS) from the network (e.g., expedited forwarding, assured forwarding), with associated parameters such as bandwidth, delay, jitter, and packet loss. A scalable and flexible QoS provisioning is discussed in [141] based on a bandwidth broker (BB) to automate admission control and router configuration. The BB allocates resources for each incoming flow based on the requested QoS, its service level agreement, and the available resources. The performance of a WCDMA cellular system supporting teleechography services is evaluated in [88] for two types of traffic with different source coding and QoS schemes. A slow fading multipath Rayleigh channel is assumed in the paper.

A load-balancing platform based on differentiation of services that improves the application-level QoS (such as client response time, priority) in a telecare application is presented in [89]. The platform allows the coexistence of Internet services and distributed objects. QoS parameters are associated to different service classes (premium, gold, bronze, and best effort). Some of these telecare services (such as signal processing/conditioning, alarm generation) need high performance and availability, while some others need higher priority (such as vital signals). When a client request arrives, the system knows what QoS parameters must be provided depending on its service class.

Experts around the world believe that new demands in providing healthcare will require fundamental changes in the structure of the network and communication [90]. It is almost impossible to separate the deployment of QoS capabilities from a thorough evaluation in the context of an application (or class of applications) with the major challenge being their vast and highly variable requirements. Some compromises proposed in [91] are (i) evaluation of potential QoS mechanisms jointly in the context of physical network elements and a representative class of “next-generation” applications, (ii) the development of mechanisms which end-users can access to participate directly in the negotiation between the network capabilities and application requirements. In LAN-based networks and in the concept of next-generation Internet, there tends to be relatively few general approaches such as (i) shaping the aggregate traffic streams with simplistic regard for the needs of individual streams, or (ii) having the capability to ensure specific service characteristics for individual streams at the expense of tremendous complexity for the user and/or application to ensure the integrity of data streams.

The wide deployment of multimedia clinical applications and the use of inter- and intrahospital communication network require a specific analysis to increase healthcare services efficiency. A processing toolbox (QoSM3) for technical evaluation of QoS traffic requirements in new healthcare services based on telemedicine is proposed in [104]. The multimedia service definition and the measurement and modeling processes permit to analyze QoS requirements and to optimize application design regarding available network resources. The proposed method has been tested to evaluate real-time and store-forward medical services. In order to extract the maximum benefit of telemedicine services, it is essential to define a precise methodology to characterize the QoS requirements for the transmitted information and the management of the available network resources [105]. Three important QoS evaluation metrics are (i) efficiency, (ii) acceptability, and (iii) usefulness. In order to optimize the QoS in these telemedicine services, it is crucial to study two aspects [106–108]: the nature of the transmitted biomedical information, and the behavior of the communication networks. The variability of the resources (e.g., in mobile infrastructures) and the heterogeneity of the connections (e.g., in the Internet) require to measure and to model the intercommunication networks [109–111].

When we study the traffic modeling to offer QoS, it is essential to begin from source and network models that compose it to understand traffic dynamics, and to use that knowledge in the subsequent design. Hence, the teletraffic engineer role [112] would be the “feedback” where the measures inform about their behavior and the QoS criteria define their performance. An extended criterion consists of managing and adapting the information generated by the applications (encoders, transmission rates, compression ratios, etc.) to the available networks resources. Specifically in healthcare environments, it would allow improving the telemedicine service QoS by looking for the optimum values [113, 114].

Reducing the elapsed time between symptom onset and treatment can be of great benefit to the patient while reducing the health care costs [83]. With mobile telemedicine, valuable prehospital transport time can be used to diagnose and evaluate the patient en route.

To alleviate the problems encountered in using mobile communications, some enhancements proposed in [83] were as follows.

- *Less reliance on commercial systems integration*. This helps in rapidly identifying and solving the problems.
- *Creation of a hybrid connectivity architecture using overlapping wireless infrastructures*. Overlapping network coverage provided by competing network providers enables (a) higher aggregate data throughput and (b) improved cell-to-cell connectivity. Combining different types of wireless connectivity, including both “always-on” Internet-protocol-based connectivity and more traditional dial-up cellular communications, the resulting smart communications architecture allows to dynamically optimize the underlying communications channels for bandwidth, delay, throughput, stability, and cost.
- Bidirectional QoS control allows the receiving physician to dynamically reprioritize the data transmission to optimize bandwidth utilization and data throughput. For example, the receiving physician can alter the frequency and quality of the images transmitted or may opt to turn on or off the waveform and numerical vital signs information being sent by the patient monitor. Providing this level of control alongside a dynamic graphical QoS display provides the physician with the ability to direct QoS delivered by the system in real time.

A prerequisite for the successful deployment of m-health services for continuous monitoring of a patient’s vital signs is the QoS support (speed, accuracy, dependability of data delivery and network availability, user mobility and number of concurrent users) by underlying wireless network(s) [92] as follows.

- High delay variations (i.e., jitter) have major negative influence on the system performance. The delay variations are contributed from the physical layer mechanisms (e.g., due to 3G bearer (re)assignments) to the high-level protocol mechanisms (e.g., protocol recovery from lower-level communication errors). Within the higher layers, the jitter may be caused by the TCP retransmission mechanism together with (unnecessary) reduction of the sending rate due to the noncongestion-based message loss. Another possible reason can be delays due to the unpredictable overall-system events like resource problems or intermediary system (processing) delays. Due to the high jitter, the average end-to-end delay is much higher than that expected.
- The 3G networks have asymmetric capacity, that is, the downlink capacity is (much) higher than the uplink. However, in telemedicine, the patient/ambulance transmits high quantities of data to the health provider/hospital.
- Another problem observed is concerned with the bearer (re)assignment within the 3G network. Data transport always starts from the common bearer (which supports low bandwidth) and then gradually, based on the traffic volume, the network assigns a higher bearer (which supports higher bandwidth). The exact conditions under which the bearer assignment takes place depend on operator. Bearer changes were observed when the offered load was increasing, but random bearer changes were also observed when the offered load was stable. This bearer change mechanism/policy might cause problem (such as buffer overflow and delay variations) for the m-health service when the service offers bursty traffic to the transport system.
- The possibility to negotiate the QoS profiles that “reserve” a particular dedicated bearer for transportation of m-health related data should be explored. If the bearer assignment mechanism is known, the service can be deployed such that it benefits from the performance characteristics of the underlying transport system. Operators and manufactures should consider this fact in order to allow for a better support of forthcoming m-health services.
- Fulfillment of the reliability requirement is critical to success of mobile wireless communications in telehealth applications. However, some services do not require 100% system reliability. Moreover, there is a tradeoff between the system’s reliability and the performance. The reliable service delivery is usually ensured by means of a TCP-based underlying transport service. This reliable service delivery may result in system performance degradation, that is, significant delay variation. Therefore, the use of unreliable UDP-based underlying transport service should be explored. A study of accuracy—(i.e., data corruption probability) and dependability—related (i.e., transport system availability, data loss probability) performance characteristics of the (UDP-based) transport system is an essential subject for future research.
- Low power consumption is also an important issue to be considered in remote monitoring of mobile patients. For example, the battery of the patient PDA will typically last only a few hours in a 3G communication, whereas 24 hours battery life is expected.

In [93], an in-depth analysis on the system bottleneck and scalability performance characteristics is presented. The QoS parameters for telemedicine constitute a set of user-specified tolerable degradations of multimedia presentations that may occur due to resource limitations. These parameters can be used to quantify the presentation process from the user's point-of-view and establish network resources requirements to ensure the delivery of multimedia information with the desired quality. Interstream synchronization could be used on applications which require that all data streams must be delivered prior to their deadlines [91]. Another quality issue is the loss of telemedicine data due to limited buffer capacity at the client site. Thus random network delays and scheduling to ensure synchronization can lead to buffer overflow. However, isochronous data such as video and audio can tolerate some information loss without affecting their play-out quality. Depending on the required quality, a user can quantify the acceptable data loss for each object.

## 6. FOURTH GENERATION (4G) MOBILE TELECARDIOLOGY

In this section, we discuss the limitations of 3G wireless technology in meeting some of the demands of efficient real-time monitoring of cardiac patients. We analyze the 3G technology and also discuss the evolution and features of 4G technology that will give a magnanimous boost to telehealth monitoring applications.

When the 3G systems scenario and capabilities evolved, a lot of research work was carried out to create next-generation telemedicine systems using this technology. One such effort was carried out by the authors in [94], who proposed a test-bed based on a 3G cellular standard for the next-generation mobile telemedicine. The 3G-based testbed (12.2 Kbps ∼ 2 Mbps) had higher and wider transmission data rates than the 2G version (up to 9.6 Kbps). To demonstrate the usefulness of the testbed and the potential to improve the QoS for mobile medical applications, a simple cardiology system was designed. Because this testbed is based on 3GPP FDD mode standard, its data traffic channel has five rates 12.2, 64, 144, 384, and 2084 Kbps to be selected according to the required medical service.

There are multiple standards for 3G making it difficult to roam and interoperate across networks. Therefore, it cannot support the global mobility and service portability. 3G is based on primarily a wide-area concept. It does not support hybrid networks that utilize both wireless LAN (hot spot) concept and cell or base-station based wide area network design. 3G systems support a peak bandwidth of only a few Mbps. Researchers have recently developed spectrally more efficient modulation schemes that cannot be retrofitted into 3G infrastructure. We need all digital packet networks that utilize IP in its fullest form with converged voice and data capability.

Research on fourth generation (4G) and beyond 4G (4G+) mobile communication systems is already under way to achieve both high data rates and extended coverage of the geographical area [59, 60, 95–98]. 4G is an initiative by researchers from various universities and industry (e.g., Motorola, Qualcomm, Nokia, Ericsson, Sun, HP, NTT DoCoMo) to move beyond the limitations and problems of 3G to address future needs of a universal high-speed wireless network that will interface with wired backbone network seamlessly [99]. A comparison between features of the 3G and 4G wireless networks is provided below.

A number of spectrum allocation decisions, spectrum standardization decisions, spectrum availability decisions, technology innovations, component development, signal processing and switching enhancements, and intervendor cooperation have to take place before the vision of 4G will materialize. 3G experiences—good or bad, technological or business—will be useful in guiding the industry in this effort.

Providing mobile communication services based on new technology such as 4G technology involves more than simply proposing and proving technology—it also requires field-testing of functions and performance, standardization of technical specifications, development of mobile terminals, and construction of network facilities. A basic approach to the technical issues and system configuration involved in achieving the capabilities and performance required of the 4G system based on the research at NTT DoCoMo is described in [100].

The major design objectives of a 4G mobile communication system are (i) providing higher transmission rates (approximately 100 Mbits/s in an outdoor mobile environment and gigabit rates indoors) and larger capacity (both in terms of number of users and traffic volume) than IMT-2000, (ii) a transmission capacity (bandwidth) of at least 10 times that of IMT-2000 has to be achieved, (iii) for achieving high throughput and high-level real-time communications, it is necessary to achieve a low transfer delay time of 50 seconds, (iv) seamless connection and handoff between heterogeneous access systems, (v) since future services will all be based on IP networks, efficient transmission of IP packets over wireless connections is also a necessity and (vi) while increased capacity is also effective in lowering the bit cost, the cost per bit must be reduced to between 1/10 and 1/100 of the current levels by reducing the infrastructure equipment, operation, and construction costs.

The major technical issues for the development of a ubiquitous 4G mobile communication system are the following.

1. *High capacity and high-rate transmission*. IMT-2000 achieves a transmission rate of 2 Mbps with a 5-MHz frequency bandwidth. Furthermore, technology for transmission at approximately 10 Mbps with the same frequency bandwidth using multilevel adaptive modulation and demodulation is under development [101]. To achieve rates of 100 Mbps to 1 Gbps, larger frequency bandwidth and new transmission systems are required that are suited to high-rate transmission. A radio access system that can transmit packets efficiently with considering the importance of indoor area coverage in the future allows the use of both indoors and outdoors. To obtain the broadband frequencies for achieving high-rate transmission and meet the expected large increase in data traffic demand, new frequency bands must be considered.
2. *Lower costs.* Using a higher frequency band to achieve a higher transmission rate generally reduces the area of the cell that a base station can cover. Retaining the original coverage area therefore requires more base stations, and this increases the network cost. It is therefore necessary to expand the cell radius by using higher performance radio transmission and circuit technology such as improved modulation/demodulation techniques that can cope with a low signal-to-noise ratio, adaptive array antennas, and low-noise receivers. To further reduce the system construction and operating costs, a study of diversified entrance links that connect base stations to the backbone network, autonomous base station control technology, and multihop radio connection technology employing simple relay stations is required.
3. *Interconnection based on IP networking*. To enable international roaming, a terminal that can be configured to work with multiple systems based on software defined radio (SDR) technology [102] could be an effective way to cope with introductory periods and differences in operating frequency bands among different countries and regions. Furthermore, future mobile communication networks will be integrated with heterogeneous access methods and various kinds of cells with interconnection capabilities based on IP networking. Accordingly, interconnection and handover between such various access systems are required in addition to handover and roaming within one mobile communication system.

The system configuration for the 4G mobile communication system is as follows. (i) IP-based connection configuration: 4G systems will be configured for connection to IP networks, considering efficient transmission of IP packets, coexistence with other access systems, ease of system introduction, expandability, and other such factors. IP networks can also connect with or accommodate wireless access systems other than 4G systems. (ii) Cell classification and configuration according to the communication environment: the 4G system has cells for outdoors, indoors, and inside moving vehicles. Outdoor cells cover a wide area, unlike the hotspot areas of wireless LANs, and allow high-rate packet transfer for fast-moving terminals. Indoor areas are covered by indoor access point (APs), because the radio waves to/from outdoor base stations suffer large attenuation. Indoor APs are designed not only to provide a high rate transfer and simple operation, but also to compete with expected future wireless LANs [142, 143]. A multihop connection, which is effective in expanding the cell size is being investigated as a way to overcome dead spots caused by shadowing. Data transmission via relay stations is expected to allow communications even when the effects of limited terminal transmitting power and radio wave propagation attenuation are large [103]. (iii) Multimedia communications: conventional IP networks have provided mainly best-effort services, but with real-time applications expected to increase as multimedia communication diversifies, the importance of services that take into account QoS is also expected to increase. The 4G system configuration allows for a mechanism that guarantees the transmission rate to some extent and that prioritizes packet transfer by the packet type in cooperation with the IP network for QoS-aware packet transmission on a mobile radio link, which is the bottleneck.

## 7. NEXT-GENERATION (xG) TELECARDIOLOGY NETWORK ARCHITECTURE

**Figure 1:**
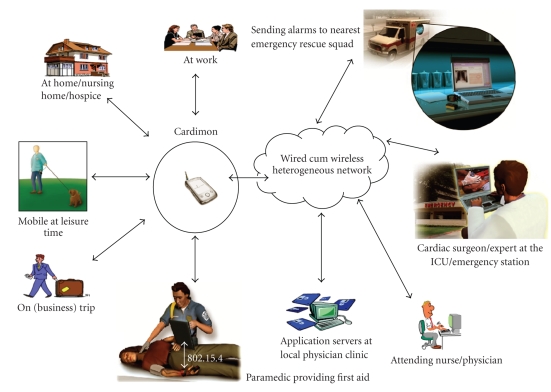
Ubiquitous monitoring of patients using xG networking.

Next-generation cardiac monitoringsystem provides ubiquitous monitoring to patients wherever and whenever necessary as shown in Figure 1. It consists of wearable and light-weight wireless biomedical sensors (for measuring 3 lead ECG, Spo2, heart beat, and blood pressure) which constantly communicate to the monitor, a unit about the size of a mobile phone or PDA. The monitor is battery powered and equipped with signal processing/conditioning module, memory, and different wireless interfaces and radios. Major features of xG system are the following.

- The signal processing module carries out local analysis of recorded physiological measurements and transmits them based on the patient-specific monitoring thresholds and response parameters, such as timing and frequency of response, as predetermined by the health provider.
- When the monitor detects an abnormal or arrhythmic event, it automatically transmits the ECG and other measurements to the health provider in real-time (with an alarm signal, if required) as discussed below. Or, when a patient experiences symptom(s), he/she can transmit the physiological measurements, symptom(s), and activity data through a monitor touch-screen.
- The system is equipped with multiple radios and the wireless interfaces for connectivity to different wireless networks such as 3G cellular, WLAN, WiMax, and wireless ad hoc (for multihop relaying). This enables the system to connect to the best available network depending on various QoS parameters.
- Location-based services using Global Positioning System (GPS) for positioning, geographic information systems and location management functions. This would enable the user to access nearby emergency services whenever required.
- Generation of alarm signals when (a) abnormal medical situation is detected, (b) health provider sends instructions to the patient or needs additional information from the patient, (c) when the system is unable to contact the health provider and send data, (d) when the system is unable to detect signals from patient’s sensors, and (e) when the system is low on battery.

The cardiac patients in xG systems could be divided into the following categories based on the severity of their health conditions as detected by the system based on the thresholds set by health provider. The health provider while prescribing the system to the patient assigns a particular class to the patient. This class is automatically changed by the transport protocol in the system depending on the severity of the health condition as follows.

- *Class 0* (highest priority requiring real-time monitoring). Patients in emergency conditions like the patients in the ambulances or in the following medical conditions:
  - ventricular fibrillation (VF), ventricular tachycardia (VT) with hemodynamic compromise, electromechanical dissociation (cardiac standstill) indicating risk of sudden cardiac death requiring medical assistance in less than 3 minutes;
  - stable VT with no hemodynamic compromise but still indicating very high risk of sudden cardiac death requiring medical assistance in 15–20 minutes;
  - AF with fall in BP and SpO2 indicates that ventricle is also beating fast, which is life threatening. Arrhythmias (such as short runs of VT) that precede and succeed a heart attack (i.e., myocardial infarction) and also precede sudden cardiac death requiring medical assistance in 30 minutes.
- *Class 1* (requiring near real-time monitoring within few hours). The patients experience the following: abnormal rhythms, such as atrial flutter, paroxysmal atrial fibrillation, fast supraventricular tachyarrhythmia, sinus bradycardia like sick sinus syndrome or mobitz type 2 block.
- *Class 2* (requiring periodic monitoring such as twice daily).
  - Patient with recurrent episodes of AF of more than 10 minutes for 4-5 times in a day who is not covered by anticoagulant like “comudin.” Health provider should be notified so that he/she can be put on blood thinning drug “comudin” to prevent development of highly morbid and often mortal disease, such as stroke.
  - Patient with recurrent and prolonged AF (frequency and duration?) associated with fast ventricular rate (150–200). Health provider should be notified for change in treatment to prevent development of sudden cardiac death.
- *Class 3* (requiring monitoring from time to time).
  - Patients with heart failure who are at risk of SCD but are not candidates to get ICD implantation. Based on the monitoring results, some of such patients may be triaged to get an ICD despite not fulfilling the criteria.
  - Patients with syncope of unknown etiology, dizziness, lightheadedness, palpitations.
  - Patients with obstructive sleep apnea to evaluate possible nocturnal arrhythmias.
  - Patients requiring arrhythmia evaluation for etiology of stroke or transient cerebral ischemia, possibly secondary to atrial fibrillation.

The cardiac telehealth application has to coexist with other telemedicine, emergency, and consumer applications on public wireless networks. These applications have different bandwidth (text, audio, video), reliability, and delay requirements. Though the bandwidth utilized by cardiac telehealth data could be much less than many multimedia applications, reliable and low latency transmission of the data could become challenging in many situations such as heavy traffic load and unavailability of reliable wireless link in dead or hot spots.

**Figure 2:**
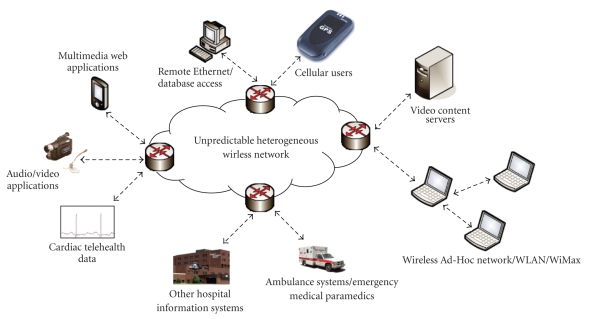
Unpredictable xG network over which the data has to be reliably sent to the remote health provider.

The unpredictable xG wireless network shown in Figure 2 could be a combination of wireless and wired networks. The wireless networks are in turn a combination of WLANs (like Wi-Fis of offices and other buildings), WiMAX networks, 3G cellular and ad hoc wireless networks.

As discussed earlier, xG system has the capability to connect to different networks simultaneously, whereas the other routers including the edge routers shown in Figure 2 may or may not have this capability. The backbone network is always chosen as the wired Internet. The front-end networks are chosen by the system based on patient’s location, required telehealth service class, and condition of available networks. The cardiac telehealth data could be sent in either a single hop or multihop manner. The multihop transfer of medical data is justified in cases of nonavailability of cellular connection in some portions of a building due to fading or interference from other networks. The data is then sent through personal area networks or Wi-Fi networks available and finally through the Internet or through a cellular base-station.

xG system first senses for the available networks and tries to obtain information about their available bandwidth, traffic characteristics, maximum delay, and so forth. As shown in Figure 2, the patient can either connect to a Wi-Fi network, which could exist in a WiMax network, or could pick up an ad hoc network. The data sent by the patient reaches the service provider through the Internet, which is the backend network or Wi-Fi or cellular network. Another critical issue of the system is that when it is unable to connect to an intermediate network, it immediately tracks a group of closely available systems (through location-based services) to whom it can directly communicate, selects the system that has the best connectivity in terms of network resources like available bandwidth, traffic through the network, and then may send the data simultaneously through all of them. This process requires tracking of neighboring systems, negotiation for cognizance of the network parameters, and selection of the best system to reduce the delay of data transfer to the nearest emergency rescue squad.

When xG system is unable to find any network for data transmission, it stores the data in its memory and transmits it as soon as it can establish connection with a network. When network connection is not available and the signal processing module detects an abnormal medical measurement, the patient may be suitably alerted by the system to contact health provider or seek medical help by locating nearby health facility with the help of GPS unit.

## 8. CONCLUSIONS

This paper provides an overview of telecardiology systems via wireless and mobile technologies. Especially, we covered 1G∼XG evolutions and their QoS requirements. There are many pitfalls encountered when creating a mobile telecardiology network. Firstly, the hardware itself must be wearable and reliable as it will be worn for long periods of time in various environments. Secondly, sensor communication to a base-station, also worn on the body, requires additional research as the interference from multiple sensors does not scale linearly. Thirdly, the communication from base-station to doctor must traverse multiple network types (3G, WiFi, 4G, Bluetooth, etc.) with different packet size requirements and data loss parameters. New protocols must be devised that allow for transmission across all networks with a consistent quality of service; because patients have differing severity of health, and sensors have different media to transmit (video, sound, text, etc.), the QoS can be different from patient to patient. In this paper, we addressed telehealth problems along with existing technologies mitigating these issues. This included the advent of xG system which can operate across a few networks with some quality of service. In sum, there are a wide variety of problems that must be overcome to make mobile telecardiology a reality.

## Figures and Tables

**Table 1:** 

Parameter	3G (including 2.5G, sub 3G)	4G
Major requirement, Driving architecture	Predominantly voice driven-data was always add on	Converged data and voice over IP
Network architecture	Wide area cell-based	Hybrid-integration of wireless LAN (Wi-Fi, Bluetooth) and wide area
Speeds	384 Kbps to 2 Mbps	20 to 100 Mbps in mobile mode
Frequency band	Dependent on country or continent (1800–2400 MHz)	2–8 GHz
Bandwidth	5–20 MHz	100 MHz (or more)
Switching design basis	Circuit and packet	All digital with packetized voice
Access technologies	W-CDMA, 1xRTT, edge	OFDM and MC-CDMA
Forward error correction	Convolutional rate 1/2, 1/3	Concatenated coding scheme
Component design	Optimized antenna design, multiband adapters	Smarter antennas, software multiband and wideband radios
IP	A number of air link protocols, including IP 5.0	All IP (IP6.0)
